# Metformin Alleviates Airway Hyperresponsiveness in a Mouse Model of Diet-Induced Obesity

**DOI:** 10.3389/fphys.2022.883275

**Published:** 2022-04-29

**Authors:** Chenjuan Gu, Jeff Loube, Rachel Lee, Shannon Bevans-Fonti, Tianshi David Wu, Jessica H. Barmine, Jonathan C. Jun, Meredith C. McCormack, Nadia N. Hansel, Wayne Mitzner, Vsevolod Y. Polotsky

**Affiliations:** ^1^ Division of Pulmonary and Critical Care Medicine, Department of Medicine, Johns Hopkins University School of Medicine, Baltimore, MD, United States; ^2^ Department of Environmental Health and Engineering, Johns Hopkins Bloomberg School of Public Health, Baltimore, MD, United States; ^3^ Section of Pulmonary, Critical Care, and Sleep Medicine, Department of Medicine, Baylor College of Medicine and the Center for Innovations in Quality, Effectiveness, and Safety, Michael E. DeBakey VA Medical Center, Houston, TX, United States

**Keywords:** obesity, asthma, insulin resistance, metformin, mouse model

## Abstract

Obese asthma is a unique phenotype of asthma characterized by non-allergic airway hyperresponsiveness (AHR) and inflammation which responds poorly to standard asthma therapy. Metformin is an oral hypoglycemic drug with insulin-sensitizing and anti-inflammatory properties. The objective of the current study was to test the effect of metformin on AHR in a mouse model of diet-induced obesity (DIO). We fed 12-week-old C57BL/6J DIO mice with a high fat diet for 8 weeks and treated them with either placebo (control, *n* = 10) or metformin (*n* = 10) added in drinking water (300 mg/kg/day) during the last 2 weeks of the experiment. We assessed AHR, metabolic profiles, and inflammatory markers after treatments. Metformin did not affect body weight or fasting blood glucose, but significantly reduced serum insulin (*p* = 0.0117). Metformin reduced AHR at 30 mg/ml of methacholine challenge (*p* = 0.0052) without affecting baseline airway resistance. Metformin did not affect circulating white blood cell counts or lung cytokine mRNA expression, but modestly decreased circulating platelet count. We conclude that metformin alleviated AHR in DIO mice. This finding suggests metformin has the potential to become an adjuvant pharmacological therapy in obese asthma.

## Introduction

Obesity affects over 650 million adults across the world (World Health Organization, 2021) and is a major risk factor for developing comorbidities such as asthma. Beyond increasing the risk of developing asthma, obesity is associated with more frequent and severe exacerbations as well as resistance to standard asthma therapy (Peters et al., 2018). The pathogenesis of obese asthma is poorly understood, and effective treatment is lacking (Peters et al., 2018). Several physiological and immunological mechanisms have been suggested, including obesity-related mechanical effects on airway, lung, and chest wall (Mead et al., 1970; Kapsali et al., 2000; Jones and Nzekwu, 2006; Raviv et al., 2011; Bates and Dixon, 2015), non-allergic airway hyperresponsiveness (AHR) and inflammation (Kim H. Y et al., 2014; Fricke et al., 2018; Younas et al., 2019), hyperleptinemia, hyperglycemia, and insulin resistance (McKeever et al., 2005; Shore et al., 2005; Wu et al., 2019a). Each of these factors may contribute to the development or worsening of asthma in obese individuals as well as their blunted response to usual therapy.

Our group previously showed that, in mice, diet-induced obesity (DIO) caused airway inflammation with elevation of interleukin (IL)-1β levels and AHR, which resembled non-eosinophilic asthma in humans (Younas et al., 2019; Fricke et al., 2018). Compared to lean mice, DIO animals had markedly increased total resistance of the respiratory system after methacholine challenge, but not at baseline (Younas et al., 2019; Fricke et al., 2018). This form of obesity-induced AHR was abolished by caloric restriction or by daily injections of the IL-1β receptor blocker anakinra (Younas et al., 2019). Interestingly, anakinra also improved insulin resistance, evidenced by the correction of hyperinsulinemia without a significant change in glucose (Younas et al., 2019). These findings suggest a potential role of insulin resistance in obesity-related AHR.

Metformin is a first-line drug widely prescribed for the management of type 2 diabetes. Metformin suppresses hepatic glucose output and increases insulin sensitivity leading to reductions in circulating glucose (An and He, 2016; Rena et al., 2017). Beyond its glucose-lowering effect, metformin has shown anti-inflammatory properties in different disease models (Saisho, 2015; Vaez et al., 2016; Hyun et al., 2013). Several retrospective cohort studies in patients with diabetes revealed a lower risk of incident asthma and better asthma-related outcomes in metformin users than non-users (Li et al., 2016; Chen et al., 2017; Wu et al., 2019b), suggesting a potential therapeutic role of metformin in asthma. However, evidence of clinical benefit is limited by the lack of randomized controlled trials. Metformin was shown to reduce airway inflammation and remodeling in allergic murine asthma models (Park et al., 2012; Calixto et al., 2013; Guo et al., 2021). By contrast, metformin did not affect AHR in studies of *db/db* mice or obese Swiss mice induced by overfeeding (Shore et al., 2008; Dias et al., 2018). Effects of metformin on airway hyperresponsiveness or inflammation in DIO mice in the absence of allergic sensitization have not been examined. We hypothesized that metformin treatment can alleviate non-allergic AHR in diet-induced obesity. In the current study, we tested our hypothesis in C57BL/6J mice on a high-fat diet.

## Materials and Methods

### Animals and Study Design

The study was approved by the Institutional Animal Care and Use Committee of the Johns Hopkins University and complied with the American Physiological Society Guidelines for Animal Studies. In total, 20 C57BL/6J male DIO mice (Jackson Labs Bar Harbor, MA, 12 weeks old) were used in the study. Mice were housed in 4 or 5 per cage, in a temperature and humidity-controlled room with a 12/12 light/dark cycle (9 am–9 pm lights on/9 pm–9 am lights off) with free access to water. The experiment consisted of 2 groups, metformin group (*n* = 10) and control group (*n* = 10). Both groups were fed with high fat diet (TD 03584, Teklad WI, 5.4 kcal/g, 35.2% fat, and 58.4% kcal from fat) *ad libitum* for 8 weeks. During the last 14 days of the experiment, metformin group was treated with metformin added in drinking water with a target dose of 300 mg/kg/day. The dose of metformin and the time course were chosen based on the previous studies (Shore et al., 2008; Dias et al., 2018). Metformin hydrochloride (PeproTech, NJ) was dissolved 3.375 g/L in drinking water, calculated based on the average body weight of mice and an estimated water consumption of 4 ml/mouse/day as Dias et al. (Dias et al., 2018). Control group was drinking water without metformin. The two groups were weight-matched before the start of metformin or placebo treatment. The body weights, food and water intake of the animals were monitored weekly. Daily food consumption was calculated by weighing food and measuring water twice a week in each cage and then divided by the number of animals and days. The food was measured both in the feeder and in the cage (given the difference in color and consistency of high fat food with bedding and feces, this was easily achieved). Cages were checked frequently, water bottles changed weekly, and no metformin-water spillage occurred during the experiment. Blood glucose levels were measured at 3:30 p.m. after a 6-h fasting (9:30 a.m.—3:30 p.m.) by tail-snip technique using a hand-held glucometer (FreeStyle Freedom Lite, Abbott, CA) once every 2 weeks.

### Physiological Measurements and Harvest

After 8 weeks’ experiment, mice were anesthetized with ketamine/xylazine i. p. and depth of anesthesia was confirmed by negative toe-pinch response before tracheostomy with an 18G stub cannula. Total resistance of the respiratory system (Rrs) was measured by forced oscillation technique (Flexivent, SCIREQ Québec, Canada) at baseline and after methacholine aerosol challenge at 3 and 30 mg/ml as described (Bishai and Mitzner, 2008; Fricke et al., 2018). Briefly, each animal was ventilated (Flexivent; Scireq, Montreal, PQ, Canada) in the supine position after injection with 5 mg/kg succinylcholine I.M. with a tidal volume of 0.25 ml of 100% oxygen at a rate of 150 breaths/min, with a positive end-expiratory pressure of 3 cmH_2_O. A deep inspiration (to 27 cmH_2_O for 10 s) was given, and then the animal was returned to normal ventilation. One minute later, a sinusoidal oscillation at 2.5 Hz was applied to determine baseline dynamic resistance (*R*rs) and elastance (*E*rs). Following the perturbation, the mouse was returned to normal ventilation for 1 min. This cycle was repeated after every methacholine dose, and average values for each dose were reported. Consistent nebulization was achieved using a 50% duty cycle for 10 seconds under normal ventilation parameters (Ultrafine Aeroneb; Aerogen, Galway, Ireland). The nebulizer was flushed between each mouse with 30 ml of water and dried to clear any residual methacholine. Blood was collected from the aorta. The thorax was opened, and the right lung was tied off, dissected free and immediately frozen in liquid nitrogen and stored at −80°C. The remaining left lung was inflated with formalin at 26 cm H_2_O pressure for 10 min, tied off and placed inflated in formalin for 2 days. Left lung volumes were measured by fluid displacement method (AU—Limjunyawong et al., 2015).

### Blood and Lung Tissue Analysis

Complete blood count (CBC) was measured by the ProCyte Dx Hematology Analyzer (IDEXX Laboratories, ME). Serum insulin, leptin and adiponectin were measured with enzyme-linked immunosorbent assay (ELISA) kits from Alpco Diagnostics (Salem, NH), Abcam (Cambridge, MA) and Millipore (Billerica, MA), respectively. Homeostasis model assessment-insulin resistance (HOMA-IR) index was calculated as fasting insulin (μU/mL) × fasting glucose (mmol/L)/22.5 for the assessment of insulin sensitivity (Matthews et al., 1985). Total RNA was extracted from lung tissue with a Trizol reagent (Life Technologies, Rockville, MD). cDNA was produced from total RNA using Advantage RT for Polymerase chain reaction (PCR) kit from Clontech (Palo Alto, CA). Real time PCR was performed for the cytokine panel, including interleukins (IL) 1β, 5, 6, 10, 13, 17, 18, and TNF-α with premade primers from Invitrogen (Carlsbad, CA) and Taqman probes from Applied Biosystems (Foster City, CA) using 18 S as a housekeeping gene. Custom made 18 S primers were forward 5′-CTC​TTT​CGA​GGC​CCT​GTA​ATT​GT-3′, reverse, 5′-AAC​TGC​AGC​AAC​TTT​AAT​ATA​CGC​TAT​T-3′ and the probe 6FAM-AGTCCACTTTAAATCCTT. Target mRNA level was normalized to 18 s rRNA, using 2^−ΔΔCt^ relative quantitative method. The results were expressed as relative fold changes to controls.

### Statistical Analyses

Statistical analyses were performed using STATA version 15.1 (StataCorp LLC, United States). All values were reported as means ± standard error of the mean (SEM). Data was checked for normality with Shapiro-Wilk W test. Normally distributed variables were compared between two groups by two-sided unpaired Student’s t-test. Non-normally distributed values were analyzed between two groups by Mann-Whitney *U* test. *p* values <0.05 were considered statistically significant.

## Results

### Basic Characteristics of the Animals

**FIGURE 1:**
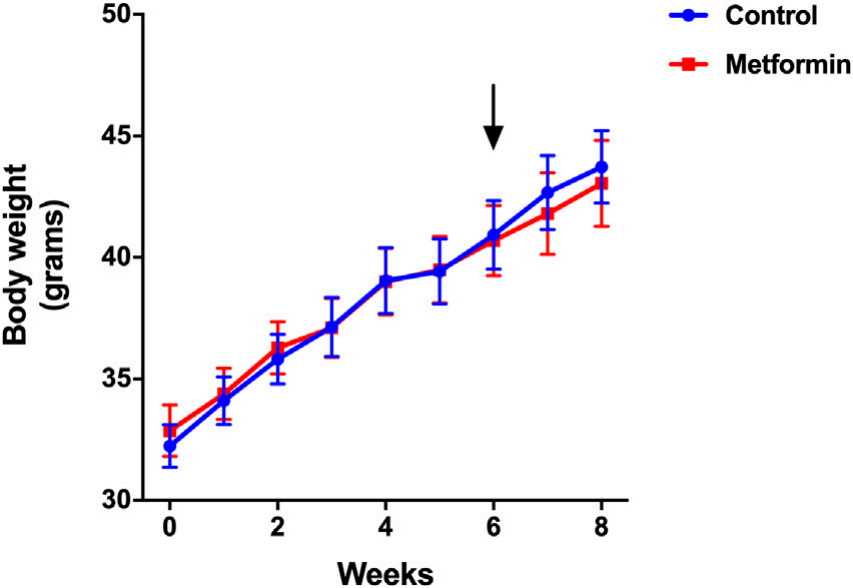
Weight trajectory of metformin and control groups over the period of 8 weeks. Data was presented as mean ± standard error of the mean. The arrow denotes the start of metformin or placebo treatment.

Mice in metformin and control groups gained similar amounts of weight over the 8-weeks study period. Body weights were identical in the two groups before and after treatments (Figure 1). Food and water intake were also similar between the two groups. The actual metformin intake was 247.8 mg/kg/mouse/day in metformin group (Table 1).

**TABLE 1:** Basic characteristics of mice in metformin and control groups.

	Metformin	Control
Number of mice	10	10
Final Age (weeks)	20	20
Final body weight (g)	43.1 ± 1.8	43.7 ± 1.5
Food intake (g/mouse/day)	2.7	2.86
Food intake (KJ/mouse/day)	60.9	64.68
Water intake (mL/mouse/day)	3.07	3.51
Metformin intake (mg/kg/mouse/day)	247.8	0

### Metabolic Profiles

**FIGURE 2:**
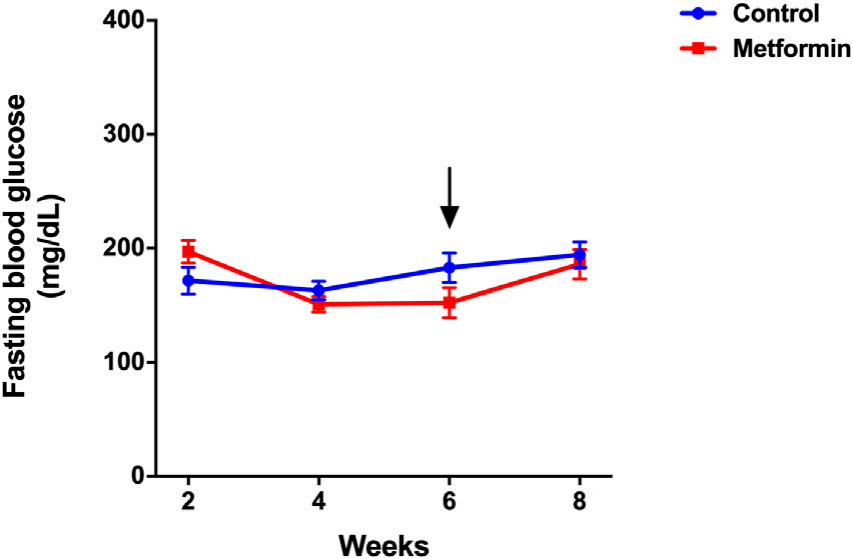
Fasting blood glucose of metformin and control groups over the period of 8 weeks. Data was presented as mean ± standard error of the mean. The arrow denotes the start of metformin or placebo treatment.

**FIGURE 3:**
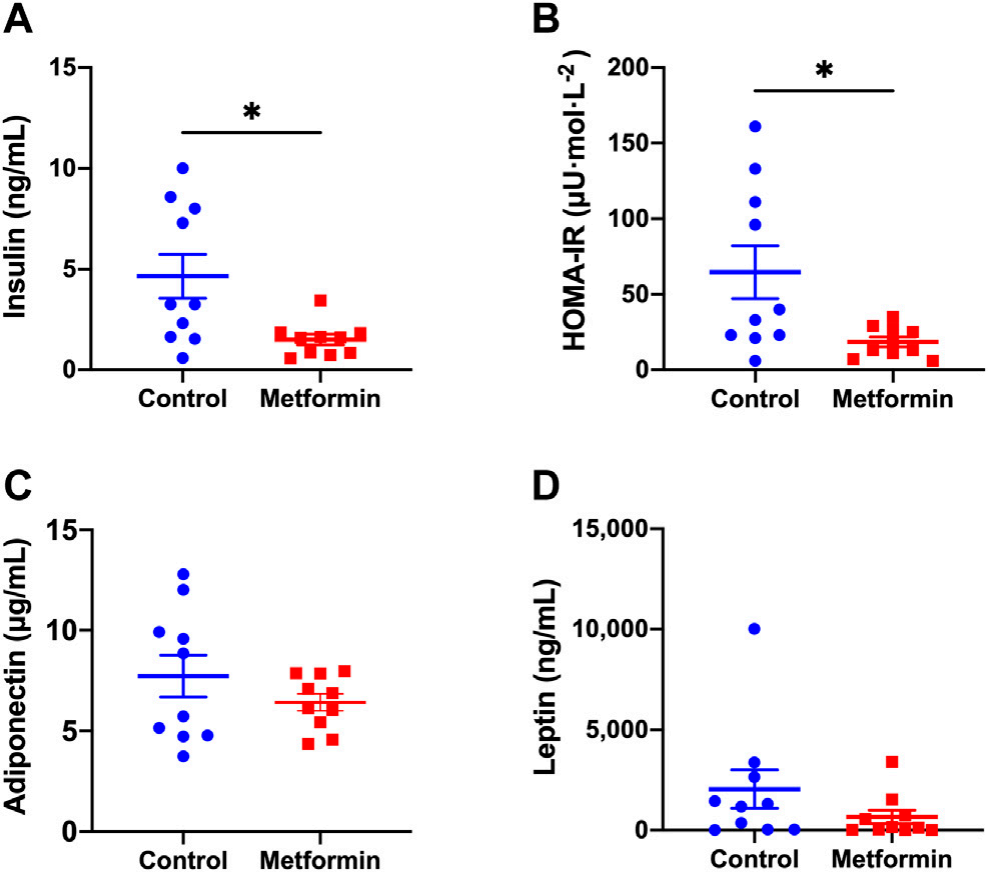
Metabolic profiles of metformin and control groups after treatments. Data was presented as mean ± standard error of the mean. * denote that these values were significantly different between two groups; Figure 3A: *p* = 0.0117; Figure 3B: *p* = 0.0179. HOMA-IR: Homeostatic Model Assessment for Insulin Resistance.

Metformin did not affect fasting blood glucose levels, which were similar between the two groups before and after treatments (Figure 2). Serum insulin and insulin resistance measured by the HOMA-IR index were significantly lower in metformin group compared to control group (Figures 3A,B; Supplementary Table S1). There was no significant difference in adiponectin and leptin levels between groups (Figures 3C,D).

### Methacholine Challenge Test

**FIGURE 4:**
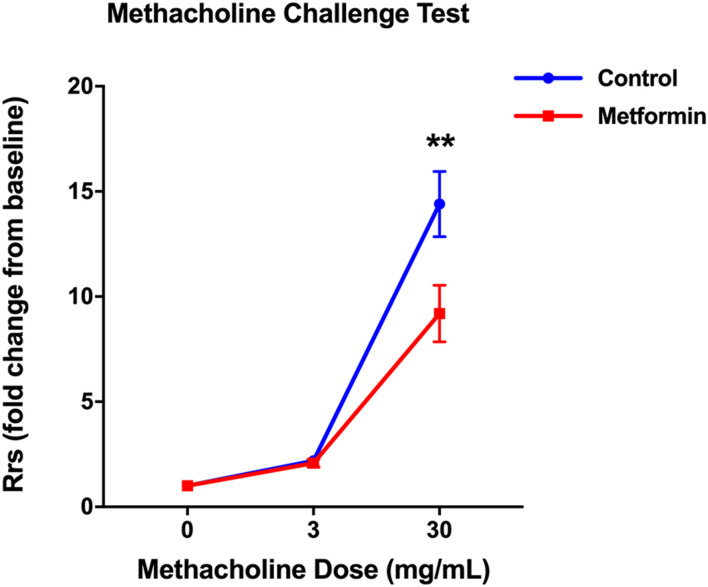
Metformin treatment decreased total resistance of the respiratory system (Rrs) in response to methacholine in diet-induced obese mice. The Rrs values were normalized to baseline (no significant difference between groups at baseline). Data was presented as mean ± standard error of the mean. ** denotes *p* < 0.01 between two groups.

At baseline, metformin had no effect on Rrs, which was 0.54 ± 0.01 cm H_2_O.s/ml in the metformin group *vs*. 0.56 ± 0.02 cm H_2_O.s/ml in the control group. After a 3 mg/ml methacholine challenge, Rrs increased to a similar extent in both groups. However, after a high dose (30 mg/ml) methacholine challenge, the metformin-treated mice exhibited lower AHR than placebo-treated mice. At this dose of methacholine, metformin-treated mice had Rrs of 5.02 ± 0.76 cm H_2_O.s/ml showing a 9.2 ± 1.3-fold increase from baseline, whereas placebo-treated mice had Rrs of 8.13 ± 0.97 cm H_2_O.s/mL showing a 14.4 ± 1.6-fold increase from baseline; *p* = 0.0052 for the effect of metformin (Figure 4). Effect of metformin on lung elastance mirrored changes in Rrs (Supplementary Table S2).

### Peripheral Blood Cells and Lung Volume Analysis

Metformin did not affect circulating white blood cell counts, but modestly decreased the platelet count compared to the control group (Table 2). Lung volumes were similar between the two groups, 0.17 ± 0.01 ml in metformin and 0.15 ± 0.01 ml in control group (*p* > 0.05).

**TABLE 2:** Complete blood count (CBC) data in metformin and control groups.

	Metformin	Control
Number of mice (n)	8	10
Red blood cells (Million/µl)	9.75 ± 0.35	10.33 ± 0.16
Hemoglobin (g/dl)	14.79 ± 0.46	15.71 ± 0.20
Platelets (K/µl)	953.6 ± 59.2^*^	1088.0 ± 30.0
White blood cells (K/µl)	5.17 ± 0.33	5.06 ± 0.30
Neutrophils (%)	7.51 ± 2.50	8.87 ± 2.17
Lymphocytes (%)	77.58 ± 2.53	79.2 ± 2.35
Monocytes (%)	8.48 ± 1.74	5.39 ± 2.17
Eosinophils (%)	1.09 ± 0.37	1.00 ± 0.31
Basophils (%)	5.35 ± 1.54	5.54 ± 1.91

*Denote that this value was significantly different between two groups, *p* = 0.047; The blood samples of 2 mice in the metformin group were clotted.

### Lung Cytokines

**FIGURE 5:**
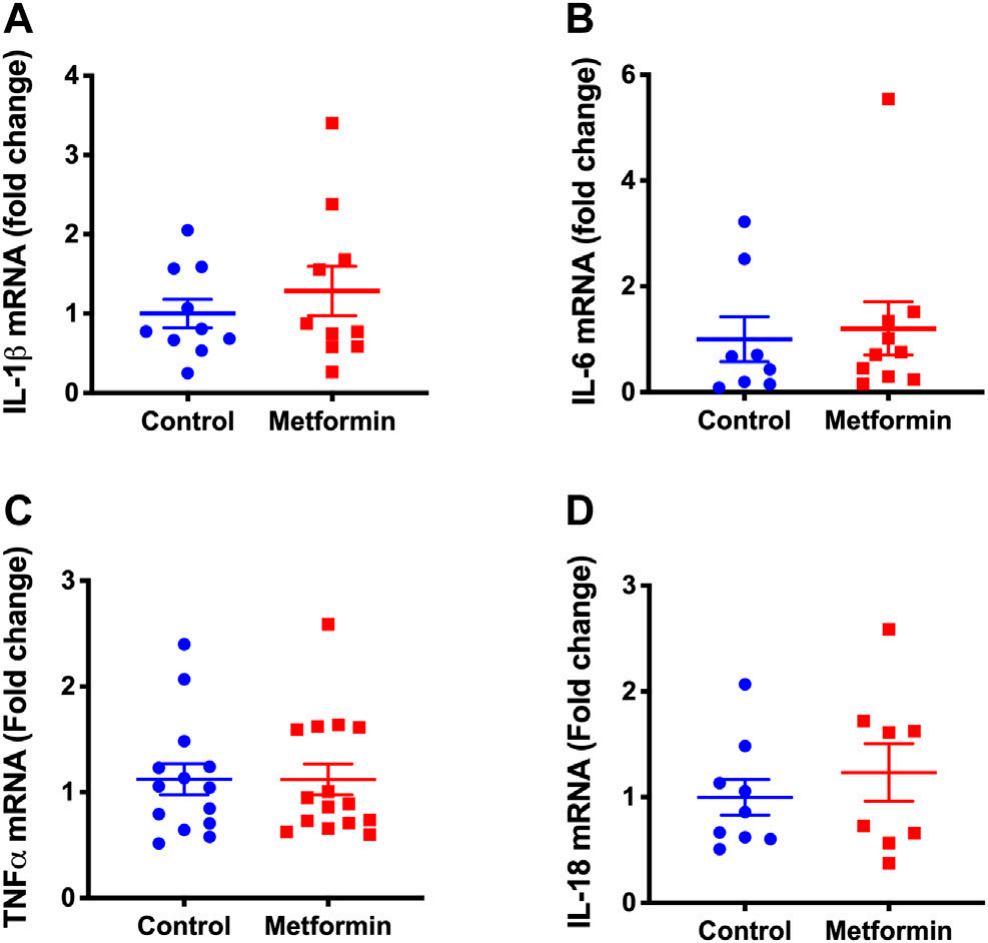
Transcription of lung cytokines in metformin and control groups after treatments. **(A)** Interleukin-1 beta (IL-1β), **(B)** Interleukin-6 (IL-6), **(C)** Tumor necrosis factor alpha (TNF-α), **(D)** Interleukin-18 (IL-18) mRNA levels in lung tissue of metformin and control groups after treatments. The results were expressed as relative fold changes to controls. Not detectable: IL-10, IL-17, IL-5, and IL-13 mRNA.

The transcription of interleukin (IL)-1β, IL-6, tumor necrosis factor alpha (TNF-α), and IL-18 in lung tissue were not significantly different between the two groups (Figure 5). Messenger RNAs of cytokines IL-5, IL-10, IL-13, and IL-17 were not detected in lung tissues of either mouse group.

## Discussion

The main finding of this study was that metformin treatment for 2 weeks alleviated AHR in a diet-induced obese asthma model. Reduction in AHR was associated with decreases in insulin resistance and the blood platelet count. In the following discussion, we address the potential mechanisms and translational implications of our findings.

AHR is a cardinal feature of asthma. The degree of AHR correlates with asthma severity, exacerbation, lung function decline, and mortality (Cockcroft et al., 1977; Brutsche et al., 2006; Leuppi et al., 2001; Becker et al., 2013). While several breakthroughs have been achieved in the treatment of eosinophilic asthma in recent years (Farne et al., 2017; Guntern and Eggel, 2020), therapy of obese non-eosinophilic asthma has not been effective. Recent human data suggest a link between obese asthma and insulin resistance (Cardet et al., 2016; Kim K.-M et al., 2014). Foer et al. provide further evidence that insulin resistance may cause or at least predispose to asthma as adult patients with asthma prescribed glucagon-like peptide-1 receptor (GLP-1R) agonists for type 2 diabetes had lower counts of asthma exacerbations (Foer et al., 2021). In diabetic cohorts, metformin users had lower risk of developing asthma, asthma exacerbations and asthma-related hospitalization than non-users (Li et al., 2016; Wu et al., 2019b; Chen et al., 2017). However, the effect of metformin on asthma has not been assessed in randomized controlled trials and mechanistic insights are lacking. A recent study showed that metformin decreases airway hyperreactivity in obese rats (Calco et al., 2021). Our experimental findings provide further evidence that metformin may be an effective treatment of obese asthma.

Studies linking metabolic syndrome to the risk of asthma and AHR in obesity have identified hyperinsulinemia as a culprit (Singh et al., 2013). In a Taiwanese diabetic cohort, insulin use increased the risk of developing asthma (Chen et al., 2017). Hyperinsulinemia in a high fat diet-induced obese rat model was associated with increased AHR to vagus nerve stimulation, which was prevented by reducing serum insulin with the pancreatic β-cell toxic agent, streptozotocin (Nie et al., 2014). Additionally, *in vitro* insulin treatment induced hypercontractility of airway smooth muscle (Nie et al., 2014; Dekkers et al., 2009), a phenotype described in asthmatic airways (Ma et al., 2002).

Our data showed that metformin corrected obesity-induced insulin resistance (↓HOMA-IR) and hyperinsulinemia independently of weight change and was associated with alleviation of AHR, supporting a role of insulin resistance in bronchial reactivity. This data is corroborated by findings by Calco *et al* which showed that male DIO rats treated with metformin had decreased bronchoconstriction induced by vagus nerve stimulation while groups treated solely with diet reversal did not experience the same reduction (Calco et al., 2021). On the other hand, our findings differ from prior studies in *db/db* mice (Shore et al., 2008) and obese Swiss mice (Dias et al., 2018) which show no effect of metformin on methacholine-induced AHR. These discrepancies could be attributed not only to strain differences, but also to the differences in metabolic profiles and methodologies for assessing AHR. *First*, *db/db* mice in Shore *et al*‘s study (Shore et al., 2008) were much more obese than the DIO mice in our study (body weight ∼60 vs. ∼ 43 g). Extreme obesity results in significant reductions in lung volumes and chest wall compliance due to mechanical effects of adiposity (Peters and Dixon, 2018). These mechanical factors augment AHR (Peters and Dixon, 2018), which is unlikely to be reduced by metformin. *Second*, *db/db* mice were severely diabetic, with ∼79 mg/dl higher fasting glucose in the control group (Shore et al., 2008) than the control mice in our study. Although metformin reduced fasting glucose by approximately 50% in *db/db* mice (Shore et al., 2008), residual hyperglycemia may also contribute to high post-treatment AHR. It is important to note that the fasting period for glucose measurement was much shorter in our study (6 h) than in Shore’s study (overnight) (Shore et al., 2008). The difference in fasting glucose between Shore’s *db/db* mice and our DIO mice could be even greater if the same period of fasting was administered. In fact, the lack of effect of metformin on fasting blood glucose in our study may represent the “floor effect”, whereas the correction of hyperinsulinemia without change in glucose suggested an improvement in insulin sensitivity. Similarly to our study, Dias *et al* reported that metformin decreased fasting insulin in obese Swiss mice without altering glucose (Dias et al., 2018). However, the relatively modest improvement in hyperinsulinemia and HOMA-IR in Dias et al. study may explain the unchanged AHR after metformin treatment. Overall, our data suggest that insulin resistance may be directly implicated in the pathogenesis of obese asthma, but molecular mechanisms remain uncertain.

Non-allergic airway inflammation is another feature of obese asthma (Kim H. Y et al., 2014; Younas et al., 2019; Fricke et al., 2018). We previously showed that diet-induced obesity in mice increased AHR in association with increased IL-1β gene expression in the lung (Younas et al., 2019) (Fricke et al., 2018) and IL-1β receptor blockade by anakinra prevented obesity-induced AHR (Younas et al., 2019), suggesting a mechanistic role of IL-1β in obese asthma. In the current study, metformin did not affect the expression of IL-1β or other pro-inflammatory cytokines in the lung. Metformin has been shown to inactivate toll-like receptor 4 and nuclear factor κB, interrupting the priming signal for the alveolar macrophage inflammasome NLRP3, an important mediator of obese asthma development in humans and mice (Kim H. Y et al., 2014). This mechanism is sufficient to suppress inflammasome-induced IL-1β activation in humans, but not in mice (Swanson et al., 2019). Metformin did not affect circulating white blood cell count and its components, but decreased peripheral platelet count in our model, suggesting a modest effect on systemic inflammation. Alternatively, metformin may affect platelets directly. In fact, metformin was shown to inhibit platelet activation and aggregation in *in vitro* and *in vivo* studies (Xin et al., 2016; Gin et al., 1989) as well as mtDNA release from platelets, an important upstream event in NLRP3 activation (Swanson et al., 2019). Platelets have been shown to actively contribute to certain features of asthma including AHR, allergic airway inflammation and airway remodeling (Idzko et al., 2015; (Takeda et al., 2018). Therefore, it is possible that metformin alleviated obesity-induced AHR through modifying the effect on platelets. Adipokine dysregulation has been shown to relate to insulin resistance (Rabe et al., 2008) and AHR (Shore et al., 2005; Coffey et al., 2015; Nigro et al., 2016). As metformin treatment did not significantly affect leptin or adiponectin levels in our model, it is unlikely that metformin improved AHR through modifying the effect of these adipokines.

Our study had several limitations. *First*, we did not measure metformin plasma levels (Chaudhari et al., 2020) and did not directly address mechanisms by which metformin improved AHR in our obese mouse model. *Second*, we did not measure pulmonary recruitment of platelets, which may play a role in obesity-induced AHR. *Finally*, we did not investigate sex differences by including female mice.

## Conclusion

Metformin reduced airway hyperresponsiveness in diet-induced obese mice. This finding suggests that metformin can be considered for adjuvant pharmacological therapy in obese asthma. Human studies are warranted to examine the translational significance of these findings.

## Data Availability

The original contributions presented in the study are included in the article/Supplementary Material, further inquiries can be directed to the corresponding author.

## Supplementary Material

Supplementary Table S1 Raw data of insulin ELISA.

Supplementary Table S2 Raw data of Methacholine Challenge Test.
